# Tumor Viruses and Their Associated Cancers: Remain on the Track with the Latest Advances

**DOI:** 10.3390/v14020262

**Published:** 2022-01-27

**Authors:** Sherif T. S. Hassan

**Affiliations:** Department of Applied Ecology, Faculty of Environmental Sciences, Czech University of Life Sciences Prague, Kamýcká 129, 16500 Prague, Czech Republic; sherif.hassan@seznam.cz; Tel.: +420-774-630-604

Infection with certain types of deoxyribonucleic acid (DNA) or ribonucleic acid (RNA) viruses, known as tumor viruses or oncogenic viruses, can lead to cancer. Studies performed in 1911 on the Rous sarcoma virus (RSV), a retrovirus that induces cancer in chickens, opened the field of tumor virology, indicating that some cancers could be triggered by specific types of viruses. Since then, extensive investigations have been conducted on tumor viruses, leading to significant findings in cancer research. Moreover, these studies have unlocked new gates for discovering strategies to prevent or manage infections with tumor viruses. Therefore, this editorial paper introduces the Special Issue “Recent Progress in Tumor Virology Research”, which has been organized to collect recent studies on tumor viruses and their linked malignancies. The Special Issue has covered interesting research papers on various tumor viruses and their associated malignancies such as human papillomavirus (HPV), canine papillomavirus (CPV), Epstein–Barr virus (EBV), a novel identified retrovirus Gunnison’s prairie dog retrovirus (GPDRV), and Merkel cell polyomavirus (MCPyV). Two topical review articles on promising therapeutic strategies for oncogenic herpesviruses and their connected cancers and HPV-associated tumors were also included. The outcome of this Special Issue might improve our understanding of the full map of the interactions between tumor-inducing viruses and the infected hosts, guiding us to finding innovative approaches to combat these viral infections and their generated tumors.

There is no doubt that infections with tumor viruses can cause serious health complications to both humans and animals. Inducing tumors is the most significant health problem reported with these viruses. Tumor viruses are categorized into two groups based on the type of nucleic acid, DNA viruses and RNA viruses [1,2]. The Special Issue “Recent Progress in Tumor Virology Research” has recently been released with several interesting studies on various tumor viruses and their associated tumors.

Multiple research groups have performed well-designed experiments on HPV, the most frequent sexually transmitted virus in the world, and its connected malignancies. Ikegami et al. [3] developed antibodies against HPV-6 and HPV-11 for the study of laryngeal papilloma, a benign tumor that is associated with HPV-6 and HPV-11 infections. In another study, McEllin and coworkers [4] demonstrated detailed genetic characterizations of HPV-16 in two oropharyngeal tumor brain metastases by targeted sequencing. Another research team detected HPV DNA using self-sampling devices in patients (women) who received long-term immunosuppressive treatment. Their study concluded that there is a need to operate a regular screening for detecting infection with HPV (as a risk factor for developing cervical cancer) in immunosuppressed women [5]. Another investigation was performed on Mexican women with gynecological alterations to diagnose the development of cervical cancer in investigated patients. The study outcome revealed that the detection of multiple HPV infections and high viral loads of various types of HPV (16, 18, 31, 35, 39, 45, 52, 56, and 59) are significant indications for recognizing patients at high risk of acquiring cervical cancer associated with chronic HPV infections [6].

CPV is a virus that belongs to the family of papillomaviruses with the ability to generate squamous cell carcinomas (SCCs) in dogs. The whole genomes of two CPV type 9 (CPV9) strains from recurrent SCCs isolated from benign or malignant skin lesions of two dogs were successfully analyzed by Chang et al. [7]. The obtained findings suggest that E2 protein and chimeric protein E8^E2 deleted CPV9 have a potential role in the oncogenesis of benign and malignant lesions; however, this role should be comprehensively explored in further experiments.

EBV, a gamma-herpesvirus (also known as human herpesvirus 4), is one of the best-examined examples of a cancer-inducing virus [8]. In a study conducted on patients with classical Hodgkin lymphoma (cHL), the prognostic function of the expression of latent membrane protein 1 of EBV (EBV-LMP1) in cHL was notably unveiled by Santisteban-Espejo and colleagues [9]. The authors also indicated that the treatment protocol based on chemotherapy medications might affect the prognostic role of EBV in cHL. In a laboratory test, the critical function of the mitochondrial enzyme glutaminase-1 (GLS1) isoforms kidney-type glutaminase (KGA) and glutaminase C (GAC) in regulating mitochondrial energy metabolism to boost EBV-infected cell proliferation was positively explored. The acquired results confirm that infection with EBV upregulated the expression of GLS1 isoforms KGA and GAC. Moreover, the results exposed a promising strategy for the treatment of EBV-associated cancers by targeting GLS1 and its isoforms with proper inhibitors [10]. The rare condition of post-transplant lymphoproliferative disorder (PTLD) linked with EBV reactivation was detected in a case report on a female patient (30-year-old) who experienced allogeneic hematopoietic stem cell transplantation (HSCT) for acute aplastic anemia. A full clinical recovery with no signs of EBV reactivation and neurotoxicity was attained by treating the patient with three cycles of a high dose of methotrexate along with rituximab [11].

Butler et al. [12] successfully identified a novel type D beta-retrovirus Gunnison’s prairie dog retrovirus (GPDRV) linked with three cases of thymic lymphoma, a type of blood cancer, in free-ranging Gunnison’s prairie dogs. The authors demonstrated that infection with GPDRV can lead to developing thymic lymphoma in Gunnison’s prairie dogs. On the other hand, the authors stated that further studies are required to isolate the newly identified virus combined with multiple experimental virological analyses.

MCPyV, a human oncogenic virus, has been observed to be accountable for the development of Merkel cell carcinoma (MCC), a lethal cancer of the skin, in humans. By employing a bioinformatics tool, MCPyV integration sites in whole-exome sequencing data from five MCC cases were entirely screened by an open access HPV Detector/Cancer-virus Detector tool. The study disclosed, for the first time, an integration that includes the tumor suppressor gene *KMT2D* [13].

This Special Issue also involves two up-to-date review articles, representing various therapeutic approaches. Berberine, a natural alkaloid compound, was introduced as a promising natural drug for treating human oncogenic herpesvirus infections and their related cancers [14]. Morgan and Macdonald [15] reviewed the mechanisms by which HPV regulates the Janus kinase/signal transducer and activator of transcription (JAK/STAT) signaling pathway, leading to active infection and promoting the development of malignancies. Additionally, inhibition of the JAK/STAT pathway has been proposed as a potential therapeutic strategy to fight against HPV-associated cancers.

Finally, all contributions that have been published in this Special Issue have delivered interesting ideas, techniques, and results, which provide a platform for other researchers to build upon.

## Data Availability

Not applicable.
